# Novel Tools and Investigative Approaches for the Study of Oligodendrocyte Precursor Cells (NG2-Glia) in CNS Development and Disease

**DOI:** 10.3389/fncel.2021.673132

**Published:** 2021-04-29

**Authors:** Christophe Galichet, Richard W. Clayton, Robin Lovell-Badge

**Affiliations:** Laboratory of Stem Cell Biology and Developmental Genetics, The Francis Crick Institute, London, United Kingdom

**Keywords:** oligodendrocyte precursor cells, NG2-glia, heterogeneity, imaging, sequencing, genetic alteration

## Abstract

Oligodendrocyte progenitor cells (OPCs), also referred to as NG2-glia, are the most proliferative cell type in the adult central nervous system. While the primary role of OPCs is to serve as progenitors for oligodendrocytes, in recent years, it has become increasingly clear that OPCs fulfil a number of other functions. Indeed, independent of their role as stem cells, it is evident that OPCs can regulate the metabolic environment, directly interact with and modulate neuronal function, maintain the blood brain barrier (BBB) and regulate inflammation. In this review article, we discuss the state-of-the-art tools and investigative approaches being used to characterize the biology and function of OPCs. From functional genetic investigation to single cell sequencing and from lineage tracing to functional imaging, we discuss the important discoveries uncovered by these techniques, such as functional and spatial OPC heterogeneity, novel OPC marker genes, the interaction of OPCs with other cells types, and how OPCs integrate and respond to signals from neighboring cells. Finally, we review the use of *in vitro* assay to assess OPC functions. These methodologies promise to lead to ever greater understanding of this enigmatic cell type, which in turn will shed light on the pathogenesis and potential treatment strategies for a number of diseases, such as multiple sclerosis (MS) and gliomas.

## Introduction

**Figure 1:**
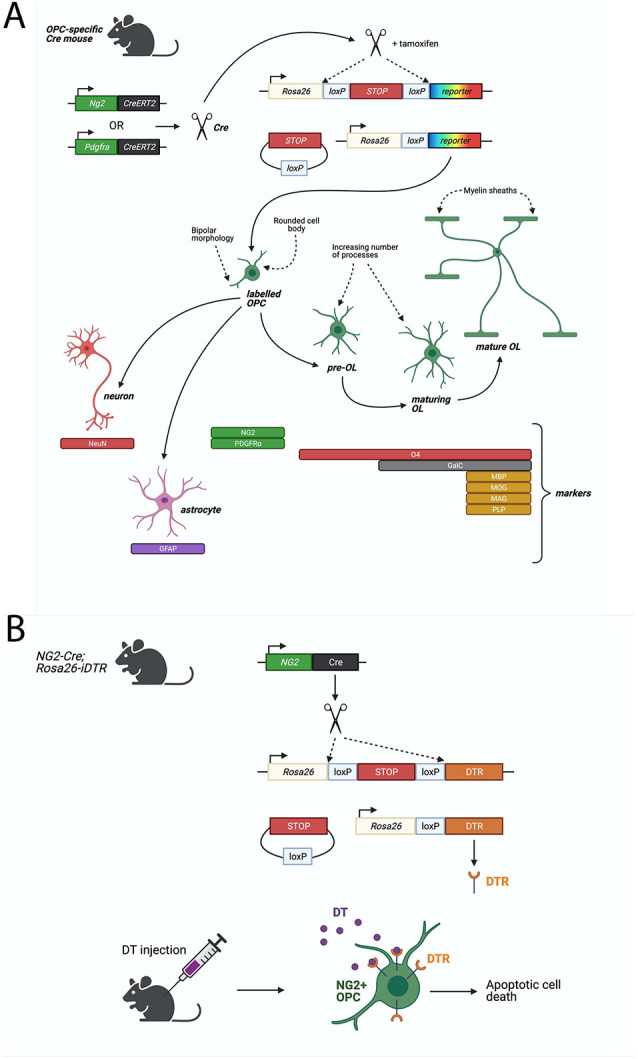
Cre-LoxP based systems to study Oligodendrocyte progenitor cell (OPC) functions. **(A)** Fluorescent proteins can be specifically expressed in OPCs in a Cre-LoxP system where Cre-recombinase or Cre-ERT2 (Cre fused to a mutant estrogen ligand-binding domain) is expressed downstream of an OPC-specific marker, such as NG2 or PDGFRα. Upon Tamoxifen administration (for Cre-ERT2), Cre-recombinase excises a floxed stop signal, which then permits expression of fluorescent protein under the ubiquitous Rosa26 promoter. Fluorescently-labeled OPCs will transmit the excised allele to their progeny. Lineage tracing of labeled-NG2-glia showed that OPCs give rise primarily to MBP^+ve^, MAG^+ve^, MOG^+ve^, PLP^+ve^ oligodendrocytes, but could also generate GFAP^+ve^ astrocytes or NeuN^+ve^ neurons. **(B)** Diphtheria toxin receptor (DTR) can be specifically expressed in OPCs by the Cre-LoxP system described above. Cre-recombinase excises a floxed stop signal, which then permits expression of DTR under the ubiquitous Rosa26 promoter. Injection of diphtheria toxin (DT) then elicits apoptotic cell death in DTR-expressing cells. In a non-inducible system, such as the one depicted, DTR will be expressed in NG2^+^ cells and their progeny which (in the case of OPCs) will result in cell death of OPCs but also any derived progeny, including oligodendrocytes. Such models must be validated for specificity of expression of DTR in the desired cell type, such as *via* immunostaining for DTR and OPC-specific markers, or by quantifying loss of OPCs and other cell types following DT-administration.

Oligodendrocytes (OLs) are the myelin-producing cells of the central nervous system (CNS) and play an essential role in facilitating neuronal signal conduction. OLs are the most numerous of the various types of glial cell in the adult mouse brain, representing approximately 20% of all brain cells (Valério-Gomes et al., 2018), and while OLs can be found throughout the entire CNS, they are most abundant in white matter tracts (Valério-Gomes et al., 2018). OLs are derived from precursor cells that can be defined by expression of the proteoglycan *NG2*, *neuron-glial antigen 2* (also known as *CSPG4*, *Chondroitin Sulfate Proteoglycan 4*; Figure 1). These progenitor cells are therefore frequently referred to as “NG2-glia,” but this term is often used interchangeably with that of “oligodendrocyte precursor cells” (OPCs), which we will use throughout this review article.

The process of oligodendrocyte lineage differentiation from OPCs, to pre-OLs, maturing OLs, and finally, fully mature OLs, has been well delineated, with many underlying transcription factors and signaling pathways having been shown to play a role (Figure 1; for review: Elbaz and Popko, 2019; Kuhn et al., 2019). In mice, the majority of OLs are generated within the first 4 weeks of post-natal life. However, generation of new OLs continues throughout adulthood (Rivers et al., 2008; Kang et al., 2010; Zhu et al., 2011), although this process declines with age, and is influenced by environmental factors such as daylight or physical exercise (for review: Wang and Young, 2014). Many excellent reviews have already extensively covered the subject of OL development (Bergles and Richardson, 2015; Traiffort et al., 2016; Elbaz and Popko, 2019; Kuhn et al., 2019; Boshans et al., 2020).

Similar to OLs, OPCs are located throughout the CNS. In the adult brain, OPCs represent approximately 5–10% of all brain cells, and are the most proliferative cell type (Dawson et al., 2000, 2003). This proliferative behavior makes OPCs especially sensitive to experimental manipulations of the cell cycle (Djogo et al., 2016). Subtypes of OPCs have been described according to various parameters, such as their spatial relation to the brain’s vasculature: e.g., perivascular, parenchymal, and intermediate (Seo et al., 2014; Maki et al., 2015; Kishida et al., 2019), whether they are located in white or gray matter, or according to the differential expression of receptors and ion channels (Spitzer et al., 2019). Morphologically, OPCs have small, rounded cell bodies (10–15 μm) and exhibit extensive branching processes. In gray matter, these processes spread out from the OPC cell body in a radial manner, whereas in white matter, they are more longitudinally orientated, following the path of axons in tracts of white matter (Levine et al., 2001). The full extent of the potential morphological, functional and molecular heterogeneity of OPCs remains unclear, as does the importance of different subsets of OPCs to brain function and disease (Zong et al., 2015; Duncan and Radcliff, 2016).

Outside of their role as progenitor cells for OLs, recent evidence has demonstrated that OPCs may have additional functions, such as interacting with other cell types and modulating their function. For example, not only do OPCs receive synaptic input from both excitatory and inhibitory neurons (Bergles et al., 2000; Lin and Bergles, 2004; Vélez-Fort et al., 2010; Nagy et al., 2017), they also synthesize neuro-modulatory factors such as prostaglandins D2 synthetase and neuronal pentrazin 2 (Sakry et al., 2015). NG2 proteoglycan, the stereotypical marker of OPCs, has itself been shown to modulate NMDA-dependent long-term potentiation and AMPA receptor sub-unit composition in excitatory neurons (Sakry et al., 2014). Moreover, targeted ablation of OPCs results in deficits in glutamatergic neurotransmission (Birey et al., 2015).

OPCs also receive inhibitory neuronal input (Lin and Bergles, 2004; Kukley et al., 2008), which, at least in the mouse cerebellum, is mediated through GABA_A_ receptors (Labrada-Moncada et al., 2020); however the functional consequence of this interaction remains unclear.

OPCs also interact with other non-neuronal cell types including astrocytes, which themselves have many roles, such as supporting and modulating neuronal function, or from regulating cerebral blood flow to maintaining the blood brain barrier (BBB; for review: Nutma et al., 2020) and microglia, which can be considered the immune cells of the CNS (for review: Domingues et al., 2016). Altogether, these findings indicate the existence and functional importance of bi-directional crosstalk between OPCs, neurons, and other cell types, and have shed light on the extent to which normal function of the adult brain may be dependent on OPCs.

It is becoming increasingly clear that OPCs are of great clinical importance, and understanding their biology and function is a prerequisite to understanding their potential role in various diseases. For example, many conditions are associated with defective myelination, such as multiple sclerosis (MS) (MS, an auto-immune disease that results in the destruction of myelin), Devic’s disease (where inflammation results in subsequent demyelination of the CNS) or exposure to chemical toxins that directly affect myelin and/or OLs (for review: Duncan and Radcliff, 2016). The formation of gliomas, including oligodendrogliomas, implicates OPCs as they represent a potential cell type from which these tumors can originate (for review: Zong et al., 2015), and the functional interaction of OPCs with neurons and other cell types may indicate their relevance for a wide range of other conditions, for example, hypopituitarism or depression (Birey et al., 2015; Djogo et al., 2016).

In recent years, our understanding of different OPC functions and characteristics has greatly expanded, and several excellent reviews have highlighted this (Dawson et al., 2000; Levine et al., 2001; Dimou and Gallo, 2015; Domingues et al., 2016; Eugenín-Von Bernhardi and Dimou, 2016; Foerster et al., 2019; Kuhn et al., 2019; Hirbec et al., 2020). In this review article, we focus our discussion on both established and novel tools and approaches that can be used to investigate the roles of OPCs in the developing and adult brain. We cover functional approaches, including use of various transgenic mice and experimental treatments, transcriptomic techniques, available imaging methodologies and physiological approaches such as electrophysiology and optogenetics, and we also discuss the multitude of culture systems used to generate and maintain OPCs *in vitro*, including 2D and 3D techniques, using primary animal or human cell lines or OPCs derived from induced pluripotent stem cells.

## Genetic Technologies

### Functional Genetics

The use of genetically altered animals offers several advantages compared to *in vitro* experiments, such as being able to study the functions of genes in an endogenous environment, with full interplay between different organ systems. Nevertheless, *in vitro* methods, which are described later in the review, bring invaluable data that often cannot be achieved using animal models. Functional genetic approaches, have revealed both intrinsic and extrinsic factors associated with different stages of OPC development; from initiation, proliferation, and maintenance to differentiation into pre-myelinating OLs and subsequently myelinating OLs (Bergles and Richardson, 2015; Elbaz and Popko, 2019; Boshans et al., 2020). For example, the use of transgenes expressing extrinsic factors have shown how several secreted factors regulate this process, including platelet-derived growth factor (PDGF; Woodruff et al., 2004), and thyroid hormone (Ahlgren et al., 1997). More recently, progesterone has been shown to up-regulate expression of *NG2*, *PDGFRα* and *Sox9* (González-Orozco et al., 2020). OPC-derived interleukin-33 (IL-33) has also been shown to regulate the differentiation of OLs to mature OLs *in vitro*, by lentivirus-mediated knock-down experiments resulting in a down-regulation of OL differentiation genes, and *in vivo*, by analyzing *IL-33* null mice which revealed defective myelination in the corpus callosum (Sung et al., 2019). Cells of the OPC lineage are also influenced by other systemic or environmental changes (ageing, inflammation or hypoxia; for review: Baydyuk et al., 2020). Extrinsic factors also include different cell types with which cells of the OPC lineage are in contact (neurons, astrocytes, blood vessels and microglia), some of which were manipulated by genetic alteration while other evidence came from co-culture *in vitro* experiments (for review: Baydyuk et al., 2020).

An especially informative genetic approach involves conditional expression of diphtheria toxin (DT) receptor (iDTR), enabling targeted ablation of OPCs in the adult mouse brain. Expression of Cre-inducible iDTR in NG2-expressing cells has allowed for the targeted induction of cell death in OPCs following DT administration (Figure 1; Birey et al., 2015; Zhang S.-Z. et al., 2019; Liu and Aguzzi, 2020). By this method, tissue-specific ablation of OPCs in the mouse prefrontal cortex was achieved by delivering DT *via* a cannula implanted into the targeted brain region, with the result of causing depressive-like behaviors, indicated by reduced open field activity (Birey et al., 2015). It was subsequently found that this loss of OPCs leads to deficits in glutamatergic neurotransmission as well as in extracellular glutamate uptake by astrocytes (Birey et al., 2015).

Similar studies, again using DT-mediated ablation of NG2-expressing cells, have provided the first indications of a role of OPCs in the maintenance of brain immune homeostasis and, while it has been known for some time that microglia can regulate the behavior and differentiation of OPCs (reviewed by Domingues et al., 2016), these studies have shown that OPCs can, in turn, modulate microglia (Zhang S.-Z. et al., 2019; Liu and Aguzzi, 2020; Marsters et al., 2020; Mecha et al., 2020). In the absence of NG2-positive OPCs, microglia become hypersensitive to lipopolysaccharide (LPS) injection, resulting in microglial activation and neuroinflammation (Zhang S.-Z. et al., 2019). OPC-derived TGF-β2 was subsequently shown to suppress microglial activation *in vitro* (Zhang S.-Z. et al., 2019). Building on this work, a more recent study has shown that, following ablation of OPCs *in vivo*, using the described DT methods in *PdgfRα^CreERT^* mice (Liu and Aguzzi, 2020), there is a whole-brain reduction in the expression of genes associated with microglial homeostasis (such as *Tmem119* and *Olfml3*). Similar effects on microglial gene expression can be observed using Crenolanib (a PDGFR inhibitor) in mouse brain slice cultures, which has the effect of depleting OPCs (Liu and Aguzzi, 2020). Finally, this role of microglial-OPC crosstalk has also been highlighted in the development of the hypothalamus (Marsters et al., 2020). Depletion of microglia, achieved by administering PLX5622, an antagonist of the receptor for the microglia-stimulating CSF1, results in decreased OPC migration to the mantle zone in the embryonic hypothalamus; a result which likely arises due to the lack of microglia-secreted cytokines (Marsters et al., 2020). Intriguingly, it is also possible that OPCs directly fulfill immune cell-associated functions, such as tissue remodeling and regulating blood vessel permeability. For example, in a mouse model of prolonged cerebral hypoperfusion, OPCs are the first cell type to produce matrix metallopeptidase 9 (MMP-9), an enzyme which degrades the extracellular matrix. Expression of MMP9 was shown to facilitate infiltration of neutrophils beyond the blood brain barrier (BBB), suggesting that OPCs may contribute to pathological neuroinflammation (Seo et al., 2013). Moreover, under certain conditions, OPCs express inflammatory cytokines such as IL-1ß or CCL-2, which are implicated in recruiting monocytes (Deshmane et al., 2009; Moyon et al., 2015). Some studies have also indicated that OPCs may be the cell of origin in gliomas/glioblastomas (Zong et al., 2015). While adult mouse OPCs were transformed by mutation of *p53* and *NF1* *in vivo* using* NG2-CreER*, which led to formation of malignant gliomas (Galvao et al., 2014), much of the molecular mechanism remains unknown.

More conventional gene mutation studies have also been instrumental in dissecting out components of OPC biology. For example, building on the potential role of OPCs in regulating the status of the BBB, tissue-specific ablation of TGF-β1 in mice (*Pdgfrα-Cre; Tgfb1^flox^*) resulted in loss of BBB function and cerebral hemorrhage (Seo et al., 2014). Other work has further refined the function of OPC-neuronal crosstalk, as previously discussed (Birey et al., 2015; Labrada-Moncada et al., 2020). From such studies, we know that OPCs express ion-channels and neurotransmitter receptors which allow them to detect neuronal activity (Larson et al., 2016) and, in turn, modulate neuronal behavior (Wang and Young, 2014; Douglas Fields, 2015; Purger et al., 2016). To give an example, the voltage-gated calcium channel (CaV1.2; Haberlandt et al., 2011) has been shown to be a major channel for depolarization-induced calcium entry in OPCs (Cheli et al., 2015, 2016). Conditional deletion of CaV1.2 within OPCs results in impaired myelination during postnatal mouse development (Cheli et al., 2016), as well as reduced reactive remyelination in a mouse model of chemically-induced demyelination (Santiago González et al., 2017). Interestingly, while studies with genetically altered mice have shown that CaV1.2 is essential for OPC survival in the adult mouse corpus callosum, it is dispensable in the motor cortex or spinal cord (Pitman et al., 2020) indicating regional heterogeneity, at least in the case of a reliance of OPCs on specific ion channels. Other conditional mutation studies have, for example, demonstrated the importance of iron storage proteins for OPC development and differentiation (Wan et al., 2020), as well as shown that Golli proteins (derivatives of the myelin basic protein gene complex) facilitate OPC migration during development by promoting Ca^2+^ transient currents in OPCs (Paez et al., 2009).

Finally, instigation of local disturbances in OPC function, through virus-mediated gene expression, has further illuminated the functional nature of neuronal-OPC crosstalk. Depending on the type of viruses used, these offer distinct approaches from affecting only dividing cells, to integrating the genetic information into the host genome, or only allowing for transient expression (Kamimura et al., 2011). For example, it is known that OPCs express AMPA receptors, activation of which by neuronal glutamate leads to OPC depolarization (Bergles et al., 2000). Retroviral delivery of various forms of modified AMPA-R GluA2 subunit to early postnatal animals (when the rate of OPC proliferation is highest; Moshrefi-Ravasdjani et al., 2017) reveals that both ionotropic and non-ionotropic properties of AMPA-R are essential for regulating the balance between OPC proliferation and differentiation (Chen et al., 2018). For example, forcible expression of a Ca2^+^-permeable AMPA-Rs in OPCs results in increased proliferation at the expense of differentiation into myelinating OLs (Chen et al., 2018). Non-virus mediated gene expression methods can also be used, such as transfection with cationic polymer or magnetofection, however, these methods currently only show low expression efficiencies (Kamimura et al., 2011).

### Lineage Tracing and Fate Mapping

Genetically-inducible fate mapping (lineage tracing allowing to follow labeled cell and their progeny) techniques have been used to study OPCs and their subsequent lineages for decades, and have made it possible to characterize and fate-map OPCs and their progeny. In embryonic mouse brains, lineage tracing experiments have demonstrated that the generation of OPCs follows a ventral-to-dorsal “Mexican wave” (Kessaris et al., 2006). While the majority of OPCs will generate OLs, recent studies have also indicated that OPCs may be capable of forming neurons and astrocytes (Rivers et al., 2008; Zhu et al., 2008a, b; Guo et al., 2010; Robins et al., 2013). Using *NG2*-Cre transgenic animals, NG2-positive OPCs were shown to generate a subpopulation of protoplasmic astrocytes in the gray matter of both the ventrolateral forebrain and spinal cord, but they did not form white matter astrocytes (Zhu et al., 2008a, b). More recent studies have demonstrated that OPCs are able to generate neurons in the adult hypothalamus, using *NG2*-CreER transgenic mice (Robins et al., 2013), and in the cortex, using *PLP-Cre^ERT^* (Guo et al., 2010) or *PdgfRα-Cre^ERT2^* mice (Rivers et al., 2008). The latter is still subject to debate, because subsequent work has indicated that OPCs do not generate cortical neurons (Kang et al., 2010; Zhu et al., 2011; Clarke et al., 2012). However, using *NG2^CreERT2^* mice, it has been shown that postnatal OPCs can generate at least some cortical neurons (Huang et al., 2014), while embryonic OPCs are exclusively gliogenic, with derivatives restricted mainly to the OL lineage, apart from a small amount of astrocyte production in the ventral forebrain (Huang et al., 2019). Tools have also been generated to study the organization and development of the OPC lineage network at single-cell resolution, notably using PLP-CreERT2 combined with the Brainbow system (Dumas et al., 2015). This method makes it possible to distinguish individual cells from their neighbors using multiple fluorescent proteins activated at random *via* the Cre/LoxP system, and further allows studies into the clonal dynamics of OPC development.

In order to characterize which neurons make direct connections to OPCs, monosynaptic tracing methods can be employed. For example, *PDGFRα-CreER* mice have been used to conditionally express the rabies virus glycoprotein 4 (gp4) and the avian TVA receptor, the receptor for subgroup A avian leukosis viruses, in OPCs. Following tamoxifen administration, gp4-deleted rabies viruses encoding GFP are stereotaxically administered to different brain regions (corpus callosum, premotor cortex). As they express the missing viral component for infection, only OPCs are infected by gp4-deleted virus allowing a monosynaptic transmission of OPC inputs to first, but not higher orders of connected cells. This revealed that corpus callosum OPCs receive brain-wide synaptic inputs both from excitatory and inhibitory neurons (Mount et al., 2019).

An important technical consideration regarding NG2- or PdgfRα-based lineage tracing of OPCs (and indeed, of any functional genetic approaches using the same gene drivers), is that NG2 and PdgfRα are also expressed by certain other cell types. These include pericytes, cells which enwrap the vasculature of the brain (Ozerdem et al., 2001), or a sub-population of adult sub-ependymal zone (SEZ) B cells and non-vascular meningeal cells (Jackson et al., 2006; Andrae et al., 2016) respectively. Lineage tracing experiments using *NG2*-CreBAC and *NG2^CreERT2^* mice may therefore encompass pericytes and any cell that is derived from them. Of course, consideration must also be given to the fact that transgenic animals may not necessarily fully reflect endogenous gene expression and/or may have ectopic expression (for review: Bouabe and Okkenhaug, 2013). One study has used a dual-promoter approach to more selectively target pericytes rather than OPCs, by requiring both expression of *Pdgfrb* and *NG2* for induction of Cre-mediated recombination (Nikolakopoulou et al., 2019). A similar approach could conceivably be used to better target and label OPCs based on the expression of multiple markers, especially given the recent studies characterizing the OPC transcriptome, which could be further interrogated to reveal novel OPC marker genes (Marques et al., 2018; Elbaz and Popko, 2019).

### Irradiation and Cell-Cycle Disruption

As mentioned above, OPCs are the most proliferative cell type in the brain (Dawson et al., 2000, 2003). OPCs are therefore especially susceptible to cell cycle disruption, and to cell death induced as a result of DNA damage checkpoints during the cell cycle (Borges et al., 2008). Consequently, inducing DNA damage through external factors [e.g., by irradiation or administration of the mitotic blocker cytosine-β-D-arabinofuranoside (Ara-C)] or genetically in the brain has the effect of predominantly affecting OPCs (Chari and Blakemore, 2002; Irvine and Blakemore, 2007; Robins et al., 2013; Djogo et al., 2016). Used concomitantly with tissue specific gene drivers, such experiments can achieve very specific ablation of OPCs. For example, Djogo et al. (2016) employed such methods to ablate NG2-glia within the hypothalamus, including AraC administration *via* the third ventricle, X-irradiation, and genetic ablation using *Sox10-iCreERT2* to delete *Esco2* (a necessary cell cycle gene). Together, these techniques demonstrated the importance of OPCs in maintaining leptin receptor-expressing neuronal processes and regulating feeding behaviors in mice. Another approach has been the use of the suicide gene herpes simplex virus thymidine kinase (*HSVtk*), expressed under the control of the *NG2* promoter in rats. Upon ganciclovir administration, dividing *HSVtk*-expressing OPCs are selectively ablated leading to impaired hippocampal neuronal functions and increased inflammation (Nakano et al., 2017). Similarly, *NG2-tk* mice have been generated to ablate NG2-positive pericytes and OPCs in the context of spinal cord injury, leading to altered astrocytic responses and recovery (Hesp et al., 2018).

The genetic techniques discussed above have been instrumental in building our current understanding of OPC biology. However, in order to further advance the boundaries of the field, more recent techniques seeking to examine OPC transcriptomes and metabolomes have begun to uncover the extent of OPC heterogeneity, and to identify novel OPC markers and potential genes for further mechanistic investigation.

## Transcriptomic Technologies

**Figure 2:**
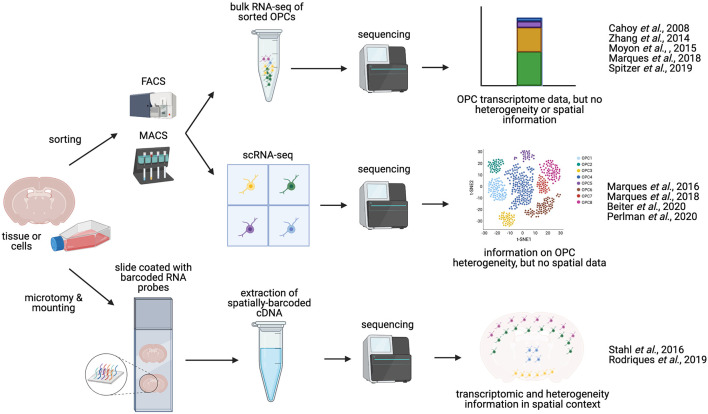
Methodologies for charaterizing OPC transcriptomics and heterogeneity. OPCs can be isolated from whole brain or spinal cord tissue. Manual dissection of different regions of the brain or spinal cord allows for anatomical comparisons. OPCs can be enriched and isolated from tissues *via* FACS (Fluorescence-activated cell sorting) or magnetic activated cell sorted (MACS, magnetic-activated cell sorting), based on expression of OPC-specific cell-surface markers, such as NG2 or PDGFRα. These sorted OPCs can then be processed and sequenced in bulk (bulk RNA-seq), or can be sequenced as single cells (scRNA-seq), yielding information on OPC heterogeneity and diversity. Alternatively, sequencing may be performed *in situ* on tissue sections [sc spatial RNA-seq or *in situ* sequencing (ISS)], producing single-cell transcriptomic data in a spatial context, and potentially mapping OPC heterogeneity to distinct anatomical regions.

The different potential functions of OPCs have begun to be revealed by mutation studies and by using techniques for genetic ablation and lineage tracing, as described. However, such advances are greatly supplemented by genome-wide transcriptomic analyses, which have become powerful investigative tools in their own right (Hwang et al., 2018). Indeed, the generation of global gene expression/regulatory networks through bulk RNA sequencing (RNA-seq) or single cell approaches has greatly refined our understanding of the biology of OPCs and OLs (Figure 2). Techniques such as single cell or single nucleus RNA sequencing (scRNA-seq and snRNA-seq respectively), where the latter allows profiling from frozen tissues or when fresh tissue dissociation is unsuccessful, while the former gives higher amounts of mRNA (Habib et al., 2017), as well as sequencing of open chromatin, which generally reflects areas of active gene expression or active enhancer regions, by Assay for Transposase-Accessible Chromatin (ATAC-seq), have been crucial in uncovering marker profiles of OPCs (Marques et al., 2018), identifying important regulatory genes and gene networks in OPC differentiation (for review: Elbaz and Popko, 2019). These sequencing methodologies have also delineated heterogeneity amongst OPC and OL populations in both spatial and temporal contexts (Marques et al., 2016, 2018; Beiter et al., 2020).

Indeed, a prevailing question that these transcriptomic techniques has begun to answer is whether OPCs and OLs represent homogenous populations, or whether they exist in functionally and transcriptomically distinct subgroups. Pioneering work, using microarray systems and bulk RNA-seq, has revealed the molecular signatures of many different cell types in the brain, including OPCs and oligodendrocytes (Cahoy et al., 2008; Zhang et al., 2014). The first study to focus exclusively on gene expression in OPCs was conducted in a mouse model of demyelination (Moyon et al., 2015). In this study, OPCs were purified through FACS, using brains from PdgfRα-GFP mice at either the neonatal stage, in 2-month old adults, or adult mice that had been treated with cuprizone for 5 weeks. Transcriptomic differences were then analyzed by microarray, and early neonatal and adult OPCs were found to differ in the expression of markers of OL differentiation, with adult OPCs having a transcriptome more akin to that of OLs than that of neonatal OPCs (Moyon et al., 2015). Interestingly, in demyelination conditions elicited by cuprizone treatment, adult OPCs reverted to a more neonatal and relatively undifferentiated state (Moyon et al., 2015).

Subsequent work has confirmed the existence of this temporal heterogeneity between OPCs in the juvenile and adult mouse brain, and further indicated that transcriptional homogeneity in the juvenile brain gives way to transcriptional divergence in the adult, which may or may not reflect regional differences (Marques et al., 2016, 2018; Beiter et al., 2020). By using flow cytometry to isolate OPCs in PdgfRa-H2B-GFP or PdgfRa-CreERT mice, followed by bulk RNA-seq and scRNA-seq, different embryonic stages were associated with district gene expression profiles that reflect the regional and temporal patterns of OPC generation. Interestingly, there appears to be a convergence of gene expression in early postnatal OPCs (Marques et al., 2018), while in the different clusters identified in adult mice, some were not specific to the OL lineage, such as fibroblasts, endothelial cells, pericytes, and leptomeningeal cells (Marques et al., 2016; Beiter et al., 2020). In the adult, OPC-specific clusters, on further investigation, were also found to be heterogenous, with genes like *Gpr17* and *Clusterin* representing novel markers of different OPC populations (Beiter et al., 2020). Furthermore, sex-specific differences in the composition of heterogenous OPC populations within specific brain regions has been recently noted, although the varieties of OPCs present throughout the brain do not appear to be different between sexes (Beiter et al., 2020). Finally, a recent study has translated these findings to humans, where scRNA-seq revealed in humans that fetal, pediatric and adult OPCs have distinct transcriptomes (Perlman et al., 2020). Furthermore, OPC diversity seems to become more pronounced over time, at least in mice (Spitzer et al., 2019).

While these data provide a valuable insight into OPC heterogeneity in the adult brain, it remains unclear whether there is a correlation between regional and functional differences in OPCs, in other words, asking whether OPCs from distinct brain regions function differently. Previous studies that targeted their scRNA-seq characterization to specific brain regions have not so far described any heterogeneity in OPCs, although it is worth noting that these studies did not focus on OPCs exclusively (Zeisel et al., 2015; Habib et al., 2016; Tasic et al., 2016; Chen et al., 2017). More recently, bulk RNA-seq of magnetic activated cell sorted (MACS) OPCs revealed that OPCs become regionally diverse with age, with respect to their expression of particular ion channels and sensitivity to neuronal activity (Spitzer et al., 2019). Since then, two molecularly distinct OPC populations have been described in the adult suprachiasmatic nucleus, but no further characterization has yet been undertaken (Wen et al., 2020). In the embryonic zebrafish spinal cord, at least two spatially and functionally distinct OPC subgroups have been described: one population that migrates laterally and generates myelinating OLs, and another that regulates neuronal excitability (Marisca et al., 2020). Furthermore, novel transcriptomic methods such as spatial transcriptomics could be used to perform scRNA-seq in tissue sections, potentially leading to the identification of uncharacterized OPC sub-groups in different anatomical regions (Figure 2; Ståhl et al., 2016).

OPC heterogeneity has also been demonstrated regarding their responses to growth factors and cytokines. For example, gray and white matter-derived OPCs respond differently to INFγ in terms of their proliferation, differentiation and process arborization (Lentferink et al., 2018), and to PDGF in terms of their proliferation (Hill et al., 2013). Furthermore, subpopulations of adult white matter OPCs differentially respond to growth factor (PDGF-AA, IGF-1 and FGF-2) and display differential gene expression patterns (Mason and Goldman, 2002; Lin et al., 2009). In summary, RNA sequencing of adult OPCs have revealed that they are a heterogeneous population of many different cells whose individual functions remain unknown.

The changing chromatin landscape has also been analyzed during OPC differentiation (Castelo-Branco and Liu, 2020) and mutations in chromatin modifiers, such as CHD7 and CHD8, have a profound effect on the formation of OLs from OPCs (Marie et al., 2018). Given the now widespread appreciation of the relationship between chromatin status and differentiation in multiple tissues (for example in skeletal muscle; Hernández-Hernández et al., 2020), this is not a surprising finding, but it nevertheless underscores epigenetic regulation as another field of ongoing research regarding OPCs (Gregath and Lu, 2018).

Finally, while transcriptomic studies yield invaluable information about the RNA contents of specific cells, proteomics are essential in correlating transcription with actual protein expression (Vidova and Spacil, 2017). In one study, large numbers of rat OPCs were cultured and differentiated *in vitro*. The proteomic profile of these cells was then analyzed at different time points throughout differentiation. Quantitative expression data was subsequently obtained for 5,259 proteins, revealing distinct patterns of regulation. Such techniques could also be used for novel candidate protein identification (Schoor et al., 2019).

Altogether, these studies indicate that, by global or local functional genetic approaches and transcriptional analysis, valuable information arises about the crosstalk between OPCs and their neighboring cells. Such information should be put in the context in which the cells are found, namely their physical interactions with their neighbors and their anatomical location. Furthermore, as OPCs are precursor cells, understanding their fate in relation to their heterogeneity will be crucial, for example, it may be that one subpopulation of OPCs is more likely to differentiate than others. Imaging techniques, including lineage tracing, are powerful tools to begin tackling these questions.

## Imaging and Microscopic Techniques

Oligodendrocytes and OPCs, like many of the different cell types in the brain, have a complex, branched morphology and reside in a substantial three-dimensional volume (Ono et al., 2001). Efforts towards understanding OPC morphology and the precise contacts they make with their neighboring cells promises to shed light on their potential functions. Conventional histological techniques, such as immunohistochemistry have highlighted important morphological differences between gray and white matter OPCs such as, as mentioned above, the former having radially orientated processes while in the latter, the processes are aligned parallel with neuronal axons (Levine et al., 2001). Of course, histological sectioning results in the loss of important volumetric information, which can otherwise more accurately inform on important parameters such as cell size, polarity, branch number, branch length, and the number of synapses and interacting cells (Hillman, 2000). Additionally, the categorization and measurement of relevant OPC parameters could be further enhanced by the use of computational techniques, such as “deep learning” which, in the case of studies concerning astrocytes, have already been used to characterize morphological parameters in a relatively unbiased manner (Kayasandik et al., 2020). It follows that investigations regarding OPC morphology may benefit from such computational techniques (Kayasandik et al., 2020), possibly highlighting previously unseen characteristics of OPCs which may provide functional insights.

Confocal microscopy has of course been essential in demonstrating the entire morphology of OPCs and oligodendrocytes (Eftekkharpour et al., 2007; Robins et al., 2013), while recent advances have facilitated imaging of whole cells in their *in vivo* context, such as light sheet fluorescence microscopy (Adams et al., 2015), two-photon or multiphoton microscopy (Table 1; Christensen and Nedergaard, 2011; Eugenin von Bernhardi and Dimou, 2019), and expansion microscopy (Wassie et al., 2019), with optical clearing allowing visualization of ever larger samples (Yu et al., 2018; Zhu et al., 2019). Zebrafish and *Xenopus* are especially useful for live imaging studies of OPCs and their lineage by light sheet fluorescence microscopy (LSFM; Bin and Lyons, 2016) or spinning disk confocal microscopy (Wang et al., 2018) due to the transparency of their embryos (Table 1). However with advances in optical clearing (Yu et al., 2018), brain tissues for larger animals (such as mice and rats) could be analyzed by two-photon or light sheet microscopy. OPC and OLs visualization could also be performed *ex vivo* (brain slice culture; Zhou et al., 2007; Gadea et al., 2009). At least with trans-cranial two-photon microscopy, *in vivo* visualization of OPCs and their lineage descendants can also be performed (Eugenin von Bernhardi and Dimou, 2019).

**Table 1:** Imaging techniques.

Technique	Type of imaging	Applications	Major benefits	Drawbacks	Notable uses	Reference(s)
(SBF-SEM) Serial block face—scanning electron microscopy	Destructive scanning electron microscopy (serial ultramicrotome sectioning)	Studying sub-cellular structures and cell-cell interactions in 3D	High resolution (5–10 nm) through 3D volume	Destroys the sample, feasible only for a relatively small total volume (slow acquisition and large data sets)	Characterization of axon diameter and myelination status in transected mouse optic nerve [5 nm (X–Y), 50 nm (Z), total volume = 50 μm (X–Y), 100 μm (Z)]; 3D-reconstruction of normal and cuprizone-demyelinated axons in mouse corpus callosum [1 nm (X–Y), 80 nm (Z), area per “slice” = 204.54 μm × 61.36 μm]	Giacci et al. (2018) and Fischbach et al. (2019)
(FIB-SEM) Focussed ion beam—scanning electron microscopy	Destructive scanning electron microscopy (focussed ion beam ablation of block face)	Studying sub-cellular structures and cell-cell interactions in 3D	Better Z-resolution than SBF-SEM (<10 nm) due to different surface ablation method	Destroys the sample, typically smaller total volumes can be imaged than for SBF-SEM	3D reconstruction of organelles in a myelinating oligodendrocyte within mouse optic nerve [7.5 nm resolution (X–Y), 30 nm (Z), total volume = 7.72 μm (X), 5.79 μm (Y), 3.81 μm (Z)].	Schertel et al. (2013)
(CLEM) Correlative light and electron microscopy	Correlated light and electron microscopy on the same samples	EM-resolution of samples (cells and sub-cellular structures) with wider contextual information	Places nm-scale EM resolution within the wider context of cells and tissues, allows for easier localization of nano-scale structures by first identifying them through fluorescent microscopy	Requires multiple types of imaging and careful alignment of separate imaging data sets of the same sample	Combined *in vivo* multiphoton microscopy, confocal microscopy, and FIB-SEM on the same samples of mouse brain (using myelinated axons as landmarks)	Luckner et al. (2018)
(LSFM) Lightsheet fluorescence microscopy	Volumetric fluroescent microscopy (sheet of illuminating light)	Whole organism/organ imaging and tissue architecture	Non-destructive fluorescent imaging of whole animals, organs or tissues	Relatively low resolution (microns)	Mapping expansion and migration of grafted human neural progenitor cells in mouse (whole mouse brain); identification of functionally, morphologically and spatially distinct subtypes of OPC in zebrafish spinal cord (entire zebrafish larvae)	Vogel et al. (2019) and Marisca et al. (2020)
Expansion microscopy	Fluorescent imaging of artificially expanded samples to increase resolution	Imaging organelles, synapses, synaptic vesicles and cell-cell interactions	High resolution beyond capabilities of typical light microscopy objectives, due to expansion of sample (effectively tens of nm)	Lengthy expansion procedure; possible distortion of tissue architecture	Super-resolution visualization of myelinated axons in mouse hippocampus (150-μm thick slices of mouse brain)	Min et al. (2020)
Super-resolution microscopy (e.g., STED—Stimulated Emission Depletion Microscopy)	Super-resolution microscopy	Imaging organelles, synapses, synaptic vesicles and cell-cell interactions	High resolution light microscopy, achieved by limiting or negating the inherent diffraction of light	Limited volumetric information, most super-resolution techniques require post-processing of images (although not STED)	Demonstration of preferential interaction of OPCs with the nodes of Ranvier of large diameter axons in mouse CNS	Serwanski et al. (2017)
Synchrotron X-ray microtomography	Non-destructive volumetric imaging	3D imaging organs and tissues	Better resolution and contrast compared to conventional μCT	High energy X-rays means cannot image live specimens, feasible only for smaller total volumes of dissected tissue (mm-scale)	Visualization of myelinated axons and other structures in a subvolume of mouse neocortex (voxel size of approx. 1 μm^3^, mm-sized piece of tissue)	Dyer et al. (2017)
Multiphoton	Non-invasive, *in vivo* fluorescent imaging	Longitudinal or time-lapse studies in live animals or cultured organs/tissues	Live imaging and ability to image cells and structures at depth within tissues, limited photobleaching, can perform optical sectioning	Relatively slow acquisition, resolution is not superior to conventional confocal microscopy	Imaging of OPC migration from SVZ to cortex in postnatal mouse brain slices	Gadea et al. (2009)

Furthermore, volumetric electron microscopy (such as ion beam-scanning electron microscopy; FIB-SEM) promise to more extensively reveal OPC/oligodendrocyte ultrastructure as already documented in the mouse optic nerve (Schertel et al., 2013) and in the lamprey nervous system (Weil et al., 2018). Such destructive electron microscopy techniques use scanning electron microscopy (SEM) to image the surface of a specimen sample, combined with an ablation method that serially eliminates the surface layer resulting in a three-dimensional stack with nm-scale resolution. Major techniques of this variety include FIB-SEM where a particle beam is used to ablate the sample surface (Schertel et al., 2013), and SBF-SEM (serial block face—SEM), where the sample is sectioned *via* microtome between images (Giacci et al., 2018; Fischbach et al., 2019; Table 1). These techniques are especially valuable when the research question requires careful identification of discrete organelles or other cellular structures that exist in a complicated 3D space. For example, FIB-SEM has been recently used to reconstruct the shape of abnormal unfolded myelin structures in mice lacking the *Anilin* gene in oligodendrocytes (Erwig et al., 2019). Physical interactions between OPCs and neurons could also be more effectively identified, classified and mapped in 3D by volumetric EM techniques, as has already been done for astrocyte-OPC interactions (Schertel et al., 2013) and more recently for synapses between astrocytes and neurons (Kikuchi et al., 2020).

Finally, imaging techniques that are based on the spin density of protons in water, notably magnetic resonance imaging (MRI) can also be used, given that the physical properties of myelin allow for it to be well contrasted against other brain tissues by virtue of its water content (40% compared to 80% respectively) (Alonso-Ortiz et al., 2015; Watanabe et al., 2016), and of course, MRI has the bonus of being non-invasive. However, the resolution given by MRI is not sufficient to allow for visualization of cells. CRISPR/Cas9 can also be used to facilitate a variety of imaging applications (reviewed by Galichet and Lovell-Badge, 2021). CRISPR/Cas9-mediated gene editing techniques that result in the epitope tagging of proteins have been developed to target neurons, and could be adapted to analyze OPCs in greater depth (Willems et al., 2020). In the next section, we discuss how the merging of functional and imaging approaches, in which light is used to modify gene expression, can be used to refine our understanding of OPC functions.

## Functional Physiological Approaches

### Electrophysiology

A recent study has used whole-cell patch clamp recordings in brain slice cultures to investigate the electrophysiological properties of NG2 positive OPCs through development and adulthood. It was observed that OPCs acquire electrophysiological heterogeneity with age, and that differences in excitability and ion channel expression are associated with distinct functional OPC states, including naïve, proliferative, primed and quiescent (Spitzer et al., 2019). While electrophysiological recordings are informative regarding ion channel expression, synapse formation and interaction with neurons and other cells, they can also provide helpful inferences regarding OPC morphology. Capacitance, a measure of stored charge, is dependent on cellular surface area, and can therefore be measured to indicate OPC size and morphology. Using this method, it was demonstrated that OPC capacitance is greatest during the active myelination phase of murine brain development (Spitzer et al., 2019). We have discussed above data showing that functional genetic approaches have revealed that OPC behavior and crosstalk with neighboring cells is region-specific. Therefore, future experiments that manage to achieve region-specific alterations, such as ablation of OPCs or their neighboring cells, would help understand the functional implications of this spatial heterogeneity.

### Functional Imaging and Optogenetics

Novel techniques of improving our understanding of OPC functions include optogenetic, intra cellular ion signaling activity and imaging (Friess et al., 2016; Ortolani et al., 2018; Zhang M. et al., 2019; Heredia et al., 2020; Marisca et al., 2020). Optogenetics involves the use of light to modulate cells expressing a light-sensitive ion channel, and has historically been used primarily to modulate neuronal activity (Lee et al., 2020). Typically, a photo-sensitive variant of channel-rhodopsin 2 (ChR2) is used. Activation of ChR2 leads to the depolarization of the cell membrane which, in neurons, could trigger an action potential (Ernst et al., 2008). To study the importance of the transient early postnatal synaptic input from GABAergic interneurons to OPCs (Vélez-Fort et al., 2010), optogenetic methods have been used (Ortolani et al., 2018). Using *Nkx2.1*-Cre and *Parvalbumin*^Cre^, ChR2 was expressed in a subpopulation of interneurons. Upon light activation on 10-days old pups, cortical OPC proliferation and density was analyzed. However, neither were affected by the increased interneuron activity, thus supporting previous findings indicating that postnatal GABAergic activity does not affect cortical oligodendroglia (Balia et al., 2017; Ortolani et al., 2018). Optogenetics also allows the control of gene expression *via* light exposure (Yamada et al., 2020) and modulation of gene expression in OPCs *in vivo* by shining light in a region-specific manner is an approach that could be used in the future.

Calcium signaling is often involved in intracellular communication (but also in some other processes), however, this may subsequently lead to release of molecules that can affect adjacent cells. Upon entry into the cytosol, calcium ions often exert an allosteric effects on a plethora of enzymes and proteins to regulate their activity and function (Clapham, 2007). In terms of the properties or behavior of a cell, the effects of Ca^2+^ can be relatively rapid (e.g., release of a signaling molecule) or more long-term and global, e.g., *via* calmodulin or calcineurin to trigger nuclear entry of a transcription factor or phosphatase activity. As Ca^2+^ ions chelate with many chemicals, methods have been developed to measure calcium variation intra-cellularly. Typically, a fluorescent molecule is fused to a chelating agent to which Ca^2+^ can bind. Upon calcium binding, the quantum-yield of fluorescence is increased resulting in higher intensity signals where Ca^2+^ concentrations are elevated (Tsien, 1980; Grynkiewicz et al., 1985; Tsien, 1989; Dean et al., 2012). Calcium imaging and calcium signaling have been used in many contexts including the study of Schwann cells (Heredia et al., 2020) and oligodendrocytes (Zhang M. et al., 2019; Rui et al., 2020). Furthermore, using calcium imaging together with lineage tracing, single-cell transcriptomics and neuronal activity manipulation, Marisca et al. (2020) have dissected the functionality of different OPC subgroups in zebrafish spinal cord, where, as described earlier, the OPC population is heterogenous, both in spatial arrangement and in functionality (Marques et al., 2016, 2018; Foerster et al., 2019; Spitzer et al., 2019). In the zebrafish spinal cord, OPC subgroups have different degrees of calcium signaling activity, visualized using the calcium indicator GcaMP6m. Calcium imaging has revealed that, in embryonic zebrafish spinal cord, OPCs showing a higher rate of calcium signaling activity are less likely to differentiate directly (Marisca et al., 2020). Furthermore, *in vitro* and *ex vivo* studies have demonstrated that intracellular calcium, alongside intracellular sodium and potassium, influence myelin basic protein synthesis in OPCs (Friess et al., 2016).

## Culture Systems

Several approaches can be taken to grow and manipulate OPCs *in vitro*, where the primary advantages are more precisely defined conditions and greater experimental tractability. Methods such as these also allow for rapid and efficient generation of OPCs and OLs, which can then be used for therapeutic purposes as part of regenerative medicine (Egawa et al., 2016; Morales Pantoja et al., 2020), or used for further experiments, such as in drug screens to identify treatments for demyelinating diseases, glioma or CNS injuries (Badr et al., 2011; Lariosa-Willingham et al., 2016). In particular, the generation of patient-derived OPCs and OLs *via* human induced pluripotent stem cells (hiPSCs) allows for the study of cell and molecular processes underlying human disease (for review: Chanoumidou et al., 2020). Furthermore, adoption of three-dimensional culture systems has enabled the study of OPCs in contexts that better recapitulate the *in vivo* environment (Chiaradia and Lancaster, 2020).

OPCs can be obtained and differentiated from mouse epiblast stem cells (Najm et al., 2011; Lager et al., 2018), from human embryonic stem cells (hESCs) or hiPSCs (Wang et al., 2013; Kim et al., 2017; Biswas et al., 2019) or by trans-differentiation of other cell types (e.g., fibroblasts) by CRISPR/Cas9-mediated gene activation (Matjusaitis et al., 2019). OPCs can also be derived from different post-natal brain regions in mice (Yang et al., 2016) by manually dissecting the region of interest and using the “shake-off” technique to discard dead or terminally differentiated cells and cells that have different adhesion properties, namely astrocytes and microglia, thereby leaving only proliferative, adherent progenitor cells (Chen et al., 2007; O’Meara et al., 2011; Medina-Rodríguez et al., 2013). Spheres of oligodendrocyte lineage cells, also known as “oligospheres” can also be obtained from multipotent mouse or rat cortical progenitor cells (Chen et al., 2007; Pedraza et al., 2008). The composition of such cultures usually requires validation by immunocytochemistry to determine the proportions of different cell types (Vitry et al., 1999; Chen et al., 2007). In comparison, purified OPC cultures can also be derived by dissociating cells from tissues and sorting for OPC-specific markers by FACS or MACS (Barateiro and Fernandes, 2014). Furthermore, zebrafish-derived OPC cultures may be ideally suited for studying fundamental processes and for small molecule screens, and can also be co-cultured with human cells (Kroehne et al., 2017).

Differentiation of OPCs and subsequently OLs from hiPSCs or hESCs can be achieved by recapitulating the signaling processes associated with differentiation (Goldman and Kuypers, 2015), either pharmacologically by timely addition of agonist and antagonists during the differentiation process (Douvaras and Fossati, 2015), or by driving expression of key regulatory genes, such as SOX10 and OLIG2 in ESCs (Pawlowski et al., 2017), or SOX10, OLIG2 and NKX6.2 (Ehrlich et al., 2017) and SOX9 in hiPSCs (Ng et al., 2020). A combination of both pharmacological and genetic differentiation methodologies can also be used to great synergistic effect (García-León et al., 2020). As mentioned, these protocols enable the production of relatively large amounts of OPCs and OLs, which can be used therapeutically (Egawa et al., 2016; Morales Pantoja et al., 2020) or for experimentation. Xenograft experiments have shown that hiPSC-derived OPCs/OLs survive and can influence disease processes in model animals (Wang et al., 2013b; Kawabata et al., 2016; Feng et al., 2020; Ng et al., 2020). Finally, and perhaps most illuminating are studies where patient-derived iPSCs are used to make OPCs/OLs, which are then used to study disease processes (for review: Chanoumidou et al., 2020).

For example, Palizaeus-Merzbacher disease (PMD) is a rare pediatric monogenic condition affecting myelin in the CNS, which is linked to a variety of mutations in proteolipid protein 1 (PLP1). Using hiPSCs derived from different patients, a screen was developed that linked particular mutations in PLP1 with specific phenotypes, such as regarding ability to develop OPCs and form OLs, cellular morphology, and myelination capacity (Nevin et al., 2017). More recently, OLs derived from MS patient iPSCs have shed light on the importance of the extracellular milieu, indicating that OPCs in the context of MS are more sensitive to inflammatory environments, which impairs their ability to differentiate (Morales Pantoja et al., 2020). The use of hiPSC or hESC-derived OPCs/OLs, in combination with “omic” methodologies described above, provide a deeper understanding of differences in gene expression between OPCs in health and disease (Lopez-Caraballo et al., 2020; Ng et al., 2020; Chamling et al., 2021). Bulk RNA-seq of hiPSC-derived OPCs for example, has indicated transcriptomic differences between OPCs in healthy donors and MS patients. Further proteomic characterization of OPC-condition media revealed that MS-associated OPCs are likely less able to promote migration of other OPCs, which was subsequently confirmed *via* *in vitro* migration assays (Lopez-Caraballo et al., 2020).

The utility of iPSC cultures for understanding OPCs in disease is, of course, not limited to the generation of OL-lineage cells. For example, it has been shown that neural progenitor cells, differentiated from hiPSCs of MS patients, were unable to promote maturation of OPCs compared to healthy neural progenitor cells, and this was linked to expression of cellular senescence genes in MS-derived neural progenitors (Nicaise et al., 2019). Another study has shown that astrocytes derived from patient iPSCs suffering from Alexander disease, which is a rare genetic leukodystrophy that features demyelination, inhibit proliferation and differentiation of OPCs in co-culture (Li et al., 2018).

Furthermore, the culture of iPSC-derived OPCs as well as from other sources, has been advanced by the incorporation of 3D culture systems. Such methods not only increase the efficiency of generation of OPCs and OLs, but also better recapitulate the *in vivo* environment, allowing for more accurate and translational studies of their biology (Rodrigues et al., 2017). Here, we will refer to both spheroids and organoids, where spheroids are small “balls” of cells of usually one or two cell types (Birey et al., 2017), and organoids being larger accumulations of cells comprised of heterogenous cell types which more closely resemble actual tissues (Chiaradia and Lancaster, 2020). For example, 3D cultures have been made by aggregation of hiPSCs, which through timely addition of signaling pathway modifiers, generate oligodendrocyte spheroids. These 3D spheroids contain cells of the OPC lineage and myelinated neurons and thus can be used for OL development and interaction with other cells (Marton et al., 2019). One limitation of cerebral organoids has been that the emergence of myelinating OLs within cerebral organoids can take a long time; between 103 and 210 days (Madhavan et al., 2018; Marton et al., 2019). However, in a recent study, hiPSCs were simultaneously differentiated into organoids containing both neurons and OL-lineage cells through a combination of differentiation media and induction of expression of cell autonomously-acting transcription factors (Ng et al., 2020). This method resulted in rapid formation of myelinating-oligodendrocytes and production of myelinated neuronal axons at a density comparable to that of healthy human brains (Ng et al., 2020). Such neuronal/OL co-cultures also allow for direct study of the process of axonal myelination in both healthy and diseased contexts using patient-derived cells (Pang et al., 2018; Assetta et al., 2020). 3D culture systems are of course also useful in glial tumor research, where gliomas and glioblastomas cultured as either tumor-spheres or as organoids permit maintenance of 3D tumor architecture (Ogawa et al., 2018; Azzarelli, 2020) and tumor heterogeneity (Jacob et al., 2020) compared to 2D culture systems (Melissaridou et al., 2019; Ruiz-Garcia et al., 2020). Brain slice cultures are also of importance in this context. For example, time-lapse imaging of brain slices could shed light on the invasiveness and evolution of gliomas as well as the cell of origin (Fayzullin et al., 2016).

Finally, the utility of conventional 2D culture systems should not be discounted, as they are nonetheless invaluable and eminently tractable methods of examining more specific aspects of OPC biology. Indeed, many of the iPSC studies described above were conducted in monolayer cultures (Wang et al., 2013; Kim et al., 2017; Yamashita et al., 2017; Biswas et al., 2019; Assetta et al., 2020). To give some further examples, treatment of *in vitro* cultured OPCs with various endocannabinoid ligands and antagonists has highlighted the potential regulatory role of the endocannabinoid system in regulating OPCs in both health and disease (for review: Ilyasov et al., 2018). Incubation of immortalized rat cerebral endothelial cells with media conditioned with primary rat OPCs resulted in increased expression of tight junction proteins and decreased transwell permeability, indicating a role of OPCs in maintaining the BBB (Seo et al., 2014). Lastly, culture of hiPSC-derived OPCs on substrates of varying stiffness has demonstrated that OPC migration is both mechanosensitive and donor-dependent (Espinosa-Hoyos et al., 2020).

## Conclusions

Oligodendrocytes, arising from oligodendrocyte progenitor cells (OPCs) or NG2-glia, are crucial cells in the nervous system facilitating rapid electrical conductance in neurons. While originally OPCs were thought of only as the precursors of oligodendrocytes, they are also able to generate other cell types including neurons and astrocytes (Figure 1), and that they have functions of their own (Rivers et al., 2008; Zhu et al., 2008a, b; Guo et al., 2010). OPCs are fully integrated in the CNS, receiving inputs from and affecting the behavior of neighboring cells including neurons, astrocytes and microglia (Domingues et al., 2016; Nutma et al., 2020). It is important to note that the appearance of OPCs from ESCs in humans follow some similar conserved transcriptional networks but also respond differently to certain factors, such as FGF2 (Hu et al., 2009; Bribián et al., 2019). It is therefore essential to understand these differences before comparing OPCs and other OL-lineage cells between species.

While OPCs share core markers, it has become increasingly clear that adult OPCs are heterogenous in their transcriptomes (Marques et al., 2016, 2018; Beiter et al., 2020). However, it is not currently fully understood how this transcriptomic heterogeneity translates into functional heterogeneity. Furthermore, OPC location may also reflect differences in OPC functions, such as illustrated with perivascular OPCs promoting angiogenesis as described under cerebral ischemia conditions (Kishida et al., 2019). With advances in transcriptomic analysis and discovery of markers specific to sub-populations of OPCs, these cells could now be sorted according to their location and/or transcriptomic profile and ATAC-seq, bisulfite-seq or proteomic studies could be performed at a single-cell level (Kashima et al., 2020). Such methods could also be applied to analyze OPCs in disease conditions.

Advances in imaging and functional imaging have allowed a better understanding of OPC localization, differentiation and relationship with other cell types. While the neuronal connectome is fundamental to understanding information processing (Oh et al., 2014), one could think of determining the OPC-specific connectome to refine our understanding of OPC integration within the CNS. With functional imaging techniques, OPCs could be ablated or have their gene expression altered in a position specific manner; this would permit further understanding of the link between OPC location and function.

Conditions affecting the function of myelin, as well as gliomas, have profound health consequences in which OPCs most likely have an etiological role to play and could be target cells for drug discovery experiments. Indeed, in specific cases of gliomas, OPCs seem to be the cell of origin (Zong et al., 2015). A greater understanding of the heterogeneity of OPCs would permit studies to determine whether gliomas that arise from NG2-glia involve a specific sub-type(s). Such information would permit a more targeted approach to treatments. In diseases affecting myelin function, such as MS or Devic’s disease (Duncan and Radcliff, 2016), it is crucial to direct OPCs to promote regeneration and remyelination. It is also important to investigate whether, for these conditions, OPCs are not themselves contributing to disease processes. For example, multiple sclerosis is an auto-immune disease affecting myelin, and OPCs contribute to brain immunity (Domingues et al., 2016) and may act as immune responder cells (Seo et al., 2013); hence a better understanding of the potential different OPC functions may help to tackle disease response and evolution. Furthermore, as OPCs are the most proliferative cells within the CNS, one could think of directing their fate in response to specific injuries (for example, generation of neurons after stroke).

It is therefore crucial to fully understand populations of OPCs; their diversity and the different functions they may have. Increased knowledge would permit the use and/or the manipulation of these cells in different neurological contexts, including those involving aging, inflammation, cancer, or degeneration.

## Author Contributions

CG and RC wrote the initial review. CG, RC and RL-B contributed to the critical reading of the review. All authors contributed to the article and approved the submitted version.

## Conflict of Interest

The authors declare that the research was conducted in the absence of any commercial or financial relationships that could be construed as a potential conflict of interest.
